# Non‐Invasive, High‐Resolution (^1^H_2_O) Metabolic Activity Diffusion Imaging [MADI] of Rat Glioma

**DOI:** 10.1002/nbm.70232

**Published:** 2026-01-30

**Authors:** Joshua W. Schlegel, Samantha M. Holland, Felice D. Kelly, Eric M. Baker, Jared Stoller, William Packwood, Xin Li, Ramon F. Barajas, Charles S. Springer, Martin M. Pike

**Affiliations:** ^1^ School of Medicine Oregon Health and Science University Portland Oregon USA; ^2^ Department of Neurology Oregon Health and Science University Portland Oregon USA; ^3^ Advanced Light Microscopy Core Oregon Health and Science University Portland Oregon USA; ^4^ Advanced Imaging Research Center Oregon Health and Science University Portland Oregon USA; ^5^ Department of Diagnostic Radiology Oregon Health and Science University Portland Oregon USA; ^6^ Small Animal Research Imaging Core Oregon Health and Science University Portland Oregon USA; ^7^ Knight Cancer Institute Oregon Health and Science University Portland Oregon USA; ^8^ Brenden‐Colson Center for Pancreatic Care Oregon Health and Science University Portland Oregon USA; ^9^ Department of Chemical Physiology and Biochemistry Oregon Health and Science University Portland Oregon USA; ^10^ Department of Biomedical Engineering Oregon Health and Science University Portland Oregon USA

**Keywords:** brain, cancer, cell density, cell volume, DWI, metabolic activity, PET, water

## Abstract

We have recently developed a metabolic activity imaging approach entitled Metabolic Activity Diffusion Imaging [MADI] which utilizes diffusion weighted MRI to quantify k_io_, the homeostatic cellular H_2_O efflux rate constant without the use of contrast agents, thus enabling measurement in both normal and tumor brain regions. Importantly, k_io_ quantifies transmembrane water cycling, a significant proportion of which is coupled to Na^+^/K^+^ ATPase activity and associated cellular energy utilization, hence constituting a key metabolic biomarker. MADI also quantifies the cell volume (V), and cell density (ρ); these enable quantification of the k_io_V and k_io_Vρ products, which convert the k_io_ rate constant to rates of water efflux *per cell* (units: pL/s/cell) and *per tissue* (units: pL/s/uL [tissue]), respectively. Representing its first application to brain cancer, MADI was comprehensively evaluated at high field (11.75 T) in rats implanted with syngeneic RG2 brain tumors, and in non‐tumor bearing rats. ^18^FDG‐PET images were obtained the following day for comparison with a commonly utilized metabolic imaging modality. A subset of rats were subsequently treated with temozolamide and radiation. Tumor ^18^FDG‐PET SUV_max_ substantially increased while tumor k_io_V substantially decreased versus contralateral. Edematous peritumoral regions indicated substantially higher k_io_V values than contralateral. The k_io_ values tended to be moderately higher than contralateral. Similarly, the k_io_Vρ tended to increase only moderately in tumor versus contralateral suggesting that the marked glycolytic activation did not substantially increase overall tissue energy production. Furthermore, the ratio of cellular water efflux to glucose uptake (WGI), suggested that tumor glycolysis remained a minor contributor to overall energy production. Mean tumor size, MADI tumor parameters, and ^18^FDG‐PET [SUV_max_] were not significantly altered with treatment. The MADI ρ was compared with that observed from a 3D confocal histological analysis. Our study supports the potential utility of the novel cytometric and metabolic MADI parameters in cancer detection and assessment.

## Graphical abstract

We employed Metabolic Activity Diffusion Imaging [MADI] and ^18^FDG‐PET in rats with syngeneic RG2 glioblastoma brain tumors. MADI quantifies k_io_ (the cellular H_2_O efflux rate constant), cell volume (V), and cell density (ρ), without the use of contrast agents. The k_io_V product quantifies the rate of water efflux *per cell*. The k_io_V was substantially decreased in tumors and demonstrated superior image resolution compared with ^18^FDG‐PET. We conclude that MADI has potential utility for cancer assessment.

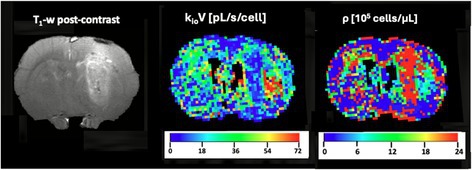

**Abbreviations**
^18^FDG: ^18^F‐labeled fluorodeoxyglucose
3D: 3‐dimensional
ADC: apparent diffusion coefficient
ApoBDs: apoptotic bodies
AQP: aquaporin
ASICs: acid‐sensing ion channels
ASL: arterial spin labeling
AWC: Active Transcytolemmal Water Cycling
Axumin: ^18^F‐fluciclovine
Bq: Becquerel
CA: contrast agent
^c^MR_AWC_
: Active Transcytolemmal Water Cycling per cell
^c^MR_AWC_
: rate of water efflux per cell
^c^MR_glc_
: rate of glucose uptake per cell
^c^MR_NKA_
: NKA flux per cell
CRT: chemoradiotherapy
DCE: dynamic contrast enhanced
DSC: dynamic susceptibility contrast
DWI: diffusion weighted image
ENaC: amiloride‐sensitive epithelial Na^+^ channel
FA: flip angle
FDOPA: 6‐fluoro‐(^18^F)‐L‐3,4‐dihydroxyphenylalanine
FET: ^18^F‐fluoroethyl‐tyrosine
fMRI: functional MRI
FOV: field of view
Glc: glucose
ID: inner diameter
*i.v.*
: intravenous
k_io_
: homeostatic [steady‐state] cellular H_2_O efflux rate constant (s^−1^)
k_io_V: rate of water efflux per cell (pL(H_2_O)/s/cell)
k_io_Vρ: rate of water efflux per tissue (pL/s/uL (tissue))
MA: metabolic activity
MADI: Metabolic Activity Diffusion Imaging
MET: L‐[Methyl‐^11^C]‐Methionine
MRSI: Magnetic Resonance Spectroscopic Imaging
NCEL: non‐contrast enhanced lesion
NHE1: Na^+^/H^+^ exchanger
NKA: Na^+^/K^+^ ATPase
NKCC: Na^+^/K^+^/2Cl^−^ co‐transporter
NMDA‐R: N‐methyl D aspartate receptor
NS: not significant
PET: Positron Emission Tomography
PO: oral
SLGL2: Na^+^/glucose cotransporter
SUV: Standardized Uptake Value
SUV_max_
: maximum Standardized Uptake Value
T_2image_
: T_2_‐weighted image
TE: echo time
^t^MR_AWC_
: Active Transcytolemmal Water Cycling per tissue
^t^MR_AWC_
: rates of water efflux *per tissue*
^t^MR_glc_
: rate of glucose uptake per tissue
TR: repetition time
TRC: tumor‐related changes
V: cell volume
vi: intracellular volume fraction
WGI: water glucose index
ρ: cell density

## 1 Introduction

For many years, water proton (^1^H_2_O) MRI has been used to diagnose and assess cancer. It is capable of impressive anatomical resolution, and its various options provide numerous strategies to effectively discriminate different tissue types. Nevertheless, its capabilities to date have remained primarily anatomical. There are some MRI‐based functional measurements; they include fMRI, which can (indirectly) detect neuronal activation, and ASL‐MRI as well as DSC‐MRI, which measure cerebral blood flow, with the latter requiring *i.v*. contrast agent (CA) injection. Vascular permeability can be assessed via DCE‐MRI, also requiring CA injection. These functional measurements, however, do not readily assess metabolic activity (MA). While cancer metabolism has been recognized as a crucial imaging goal, current options are limited. MR spectroscopic imaging (^1^H, ^23^Na, ^31^P) can detect various biological metabolites, but only within large tissue voxels, which limits its clinical utility [1, 2, 3]. Hyperpolarized carbon‐13 (^13^C) MRSI is an emerging molecular imaging method that enables pathway‐specific investigation of dynamic metabolic processes, but requires highly specialized equipment, which is only available at a few imaging centers nationwide, and also suffers from relatively poor imaging resolution [4, 5, 6]. Deuterium (^2^H) MRI is another emerging approach for assessing energy metabolism but shares the same issues as hyperpolarized ^13^C in terms of availability, use of expensive tracers, and image resolution [7, 8]. PET imaging is a widely used metabolic imaging approach, which can utilize a variety of beta‐positive emitting tracers to target different disease pathologies. However, it has relatively poor resolution, is less available than MRI, and requires radioactive tracer administration. For cancer, the primary MA imaging option employs the radioactive tracer ^18^FDG (^18^F‐labeled fluorodeoxyglucose), a non‐metabolizable analog for glucose, a key cellular energy substrate. FDG is taken up by cells through glucose transporters (GLUT1, GLUT3) and accumulates, the extent to which quantifies the tissue glucose uptake rate, an important MA biomarker. ^18^FDG‐PET imaging is used for the diagnosis and monitoring of many cancer types because tumors take up substantially increased amounts of glucose. ^18^FDG‐PET is also used in neurological disorders such as Alzheimer's Disease, Parkinson's Disease, and Epilepsy, which can show pathologic alteration of glucose uptake.

**FIGURE 1:**
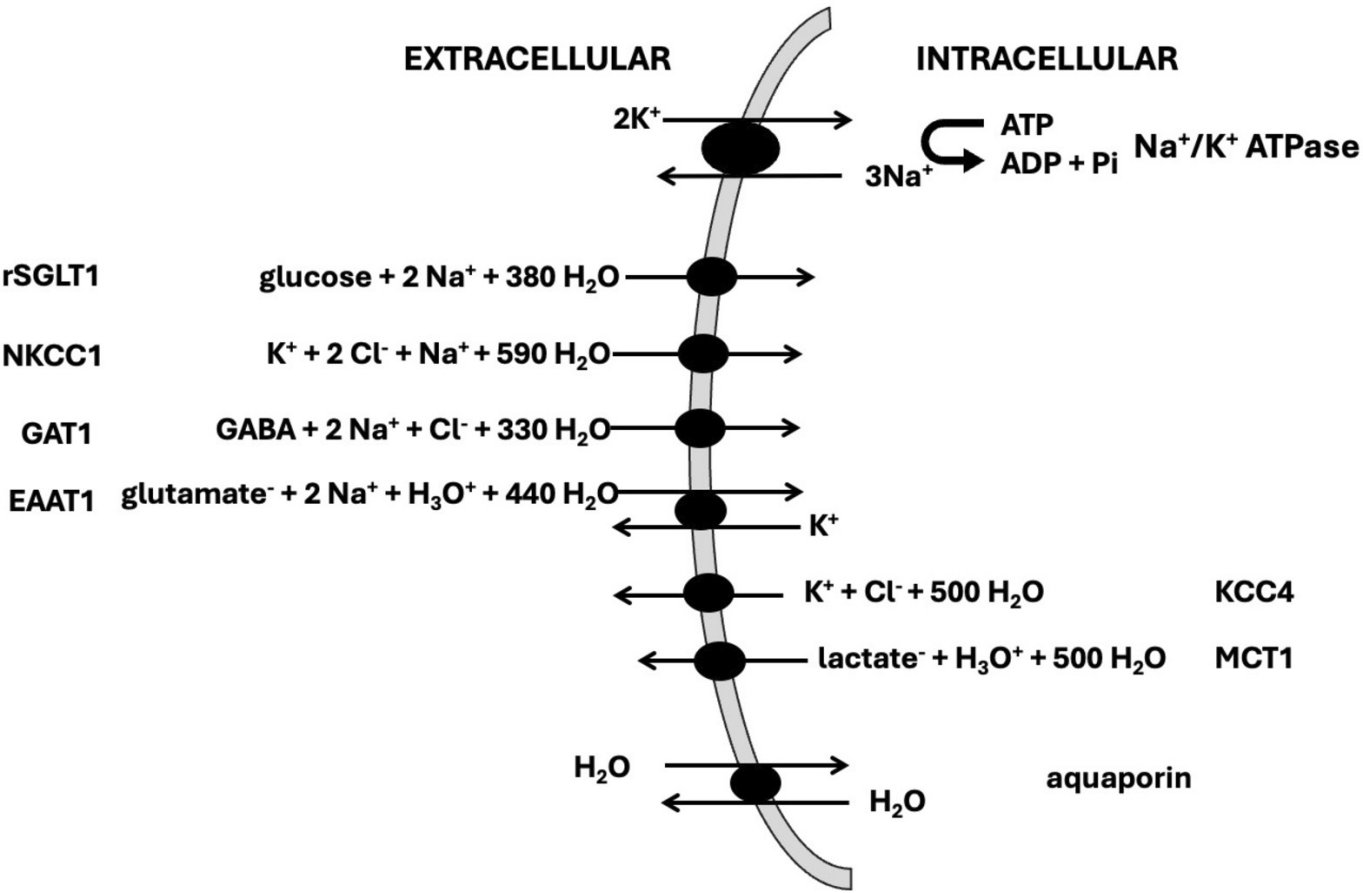
Transcytolemmal transport mechanisms common in brain are depicted, with transport stoichiometries of ions, metabolites, and water, as reported by Zeuthen (Ref. [20]).

Recently, there has been a growing realization that changes in homeostatic trans‐membrane water transport (“exchange”) constitute an important cancer biomarker [9, 10, 11, 12, 13]. To date, approaches to study this employ some variant of DCE‐MRI. Our finding that a large component of it is MA‐driven rather than passive supports the relevance of transmembrane water flux in MA imaging [14, 15, 16, 17, 18, 19]. This is related to the discovery that many of the ion and energy substrate transport processes coupled to the Na^+^/K^+^ ATPase (NKA) also actively transport water with large stoichiometries (e.g., 500 H_2_O per K^+^ for the KCC4 cotransporter), as shown in Figure 1 [20]. This Active Transcytolemmal Water Cycling (AWC) is coupled to NKA activity, the vital enzyme that maintains the trans‐membrane Na^+^ and K^+^ ion concentration gradients, which in turn drive the majority of active trans‐membrane ion and metabolite transport for the cell. The NKA (a.k.a the Na^+^ pump) accounts for a large proportion of metabolic energy consumption in most cells; in brain tissue it is estimated to be 55% [21, 22, 23].

Our recent work has shown that diffusion‐weighted MRI (DWI) is sensitive to water movement across cell membranes [17, 18]. We have developed an MA imaging approach entitled Metabolic Activity Diffusion Imaging [MADI]. MADI utilizes DWI to quantify the dynamics of this trans‐membrane water movement, in the form of the parameter k_io_, the homeostatic [steady‐state] cellular H_2_O efflux rate constant. While other CA‐based MRI methods can obtain k_io_, these have limited utility, as accurate k_io_ determination requires interstitial CA concentrations often not feasible in vivo, and precluded in normal brain tissue, due to CA blood–brain barrier impermeability [17, 18]. Importantly, k_io_ quantifies AWC, a process coupled to NKA activity and cellular energy utilization, and, hence, k_io_ constitutes a key metabolic indicator [16]. Recent reports support the link between NKA and k_io_. In rat brain slices and intact perfused rat heart titration of extracellular K^+^ over the range, which modulates NKA activity (1–8 mM) also modulates k_io_ over a large range with a pattern resembling known NKA Michaelis–Menten behavior [15, 24]. It also closely matches changes seen in O_2_ consumption measured in rat brain synaptosomes similarly titrated with extracellular K^+^, including the observed decrease when K^+^ reaches levels sufficient to induce depolarization [25]. In figure 3 of Springer et al. [19], these data (rat brain slice k_io_ and rat brain synaptosome O_2_ consumption) are plotted against each other at their equivalent extracellular K^+^ values and show a remarkable linear correlation. In both rat brain slices and in perfused rat heart marked k_io_ decreases are observed with ouabain NKA inhibition [14, 24]. Also, exposing superfused brain slices to the voltage‐gated sodium channel inhibitor tetrodotoxin or an inhibitor cocktail for excitatory postsynaptic glutamate neurotransmitter receptors blocks neuronal activation potentials and associated NKA flux, decreasing k_io_ up to 71%. In contrast, k_io_ increases with increased neuronal firing [15]. Supporting the link between energy production and k_io_ are observations in yeast that ATP depletion via hypoxia decreases k_io_ by 42% [26]. Furthermore, the inhibition of active Na^+^ transport in yeast markedly decreases k_io_ irrespective of ATP levels, indicating dependence on Na^+^ extrusion and suggesting that the k_io_ energy dependence is related to energy required for such Na^+^ extrusion [26]. Also consistent with a link between k_io_ and NKA activity is the observation that treatment of breast cancer cells with doxorubicin decreased k_io_ and also decreased NKA mediated influx of the K^+^ analog, Rb^+^ [12]. In a recent review, we have listed 19 different examples of k_io_ responses to experimentally induced metabolic changes [27]. In Springer et al. [19], we discussed the possible synchronous activity of NKA and aquaporin (AQP) in AWC, and their relationship to the hydrostatic transcytolemmal pressure gradient mechanically induced by the cell membrane. Evidence was presented suggesting that AQP largely affects water efflux in response to the hydrostatic pressure gradient and is counterbalanced by water influx via transporters driven by NKA activity, thus providing additional support for the important role that NKA plays in AWC [19].

An important advantage of the MADI approach is that in addition to k_io_, it also quantifies cell volume (V) and cell density (ρ). The intracellular volume fraction (vi) is subsequently derived: vi = Vρ product. Provision of these key cell metrics also enables quantification of the k_io_V and k_io_Vρ products, which convert the k_io_ rate constant to rates of water efflux *per cell* (^c^MR_AWC_, units: pL(H_2_O)/s/cell) and *per tissue* (^t^MR_AWC_, units: pL/s/μL (tissue)). Because a substantial proportion of this water efflux is coupled to the NKA activity, these rates are indicative of the homeostatic (cellular or tissue) NKA metabolic rate [17]. Hence, k_io_V and k_io_Vρ are related both to NKA flux and the energy it consumes, thus constituting unique and novel MA biomarkers [14, 15, 16]. The relation of k_io_V to NKA flux per cell [^c^MR_NKA_] has been formally described [18].

MADI DWI acquisition employs a standard pulsed field gradient acquisition protocol, which utilizes a large range of b values. The MADI analysis takes into account that the cell membrane is the dominant restriction to water diffusion and employs a library of simulated b‐space decay curves from a virtual ensemble of Voronoi cells to model water diffusion. It extracts more definitive information from the basic diffusion experiment than an apparent diffusion coefficient (ADC) can provide. The experimental voxel signal decays with increasing b are matched to the library of (thousands of) decays simulated for the various combinations of k_io_, V, and ρ [18]. We hypothesize that k_io_V and k_io_Vρ, which quantify AWC and by extension reflect NKA flux and associated energy utilization, in conjunction with V and ρ, are likely to be clinically useful metabolic and cytometric biomarkers. DWI data is often obtained to produce ADC maps, which are known to be sensitive to cell density, but in practice, using ADC maps to assess this has been challenging [28]. Notably, ADC values are not only influenced by cell density, but by AWC as well. The approach our team has developed untangles the variables affecting DWI images and enables cell volume and cell density to be quantified concurrent with AWC, thus providing a potentially powerful combination of parameters for cancer assessment. MADI offers several potential advantages in comparison with PET: (a) improved resolution, (b) wider availability, and (c) non‐invasiveness [no radiation dose or injection] [17, 18, 29]. The current study quantified the full complement of cytometric and metabolic MADI parameters within various brain regions in non‐tumor bearing rats, and in tumor, peritumoral, and contralateral regions in the intracerebral syngeneic RG2 glioblastoma rat model, representing its first application to brain cancer. ^18^FDG‐PET images were concurrently obtained in the same animals thus enabling a direct comparison of the modalities. Our study represents the first to comprehensively evaluate MADI utility for cancer assessment and demonstrates that MADI provides a unique metabolic assessment without the use of ionizing radiation and provides a novel modality for tumor detection. The combination of MADI MRI and ^18^FDG PET also provides an approach for evaluating the tumor aerobic glycolytic activation (Warburg effect) in the context of overall cellular energy production [30, 31, 32, 33]. Additionally, the current study is the first to compare MADI ρ values obtained in vivo with histological ρ values obtained from the same tissue.

## 2 Experimental

### 2.1 Animal Procedures

Rat studies were conducted with the approval of the Oregon Health and Science University Institutional Animal Care and Use Committee and under the supervision of the OHSU Department of Comparative Medicine. Fourteen female Long Evans rats between 3 and 6 months old were obtained from Charles River Laboratories and implanted with syngeneic RG2 rat glioblastoma cells into the right caudate nucleus at a concentration of 1 × 10^7^ cells suspended in 10 μL culture media using a stereotactic surgical apparatus. The RG2 cells were maintained in Dulbecco's Modification of Eagle's Medium (Corning, 10‐013‐CV) containing 10% fetal bovine serum and penicillin/streptomycin. Approximately 9 days later, the animals underwent MRI, followed by a 15–18 h fast and PET imaging the following day. Six of the animals were randomized to a treatment of 2Gy X‐ray irradiation to the right brain hemisphere (RadSource RS2000 X‐Ray Irradiator and 20 mg/kg Temodar (temozolomide)) 1X PO the day following PET imaging; the other animals were humanely sacrificed the day following the PET imaging. Animals that received chemoradiotherapy (CRT) were maintained in the vivarium and underwent follow‐up MRI and PET imaging 5 and 6 days post treatment, respectively, and humanely sacrificed the following day. Brains were removed, fixed in 10% formalin, sectioned at 100 μm and stored in 1X Tris with 0.05% sodium azide in a 4C fridge. Two control rats without glioma underwent identical MRI/PET imaging sessions. Of the 12 rats with tumor, one rat's data was discarded due to severe necrosis in the brain tumor, which severely degraded the quality of the MRI data. Of the six treated rats, one was euthanized prior to the second imaging session due to indications of deteriorating health. Rats were anesthetized for imaging with dexmedetomidine/ketamine (0.5/60 mg/kg, *i.p*.).

### 2.2 Confocal Microscopy and Analysis

The stored brain tissue sections were incubated in the fluorescent nuclear stain Hoechst 34580 1:800 overnight and subsequently mounted on slides with CitiFluor CFMR2 for 3D confocal imaging. Confocal images were collected from three treated and two untreated rat brains employing a Zeiss LSM900 inverted microscope controlled with Zeiss Zen 3.7. Hoechst was excited with a 405‐nm laser and emission light was collected from 400 to 600 nm on a GaASP‐PMT detector with 2× averaging. For 3D analysis of nuclear density, Z‐stacks were acquired using a Plan Apochromat 40×/1.4NA oil immersion objective. Each Z‐stack covered 20 μm in Z, with 0.19 μm Z steps and 160 × 160 μm in the XY direction. For the brains studied with histology, a single coronal section intersecting the tumor was examined in three different regions: tumor, peritumor, and contralateral; three visual fields were examined in each of the three regions. Nuclear segmentation of the Zstack was accomplished with the Cellpose version 2.0 [34] algorithm integrated into an image analysis pipeline in Zeiss Arivis Pro, version 4.30. Nuclear segmentation settings were adjusted between the tumoral area and the peritumoral and contralateral areas to improve accuracy of nuclei counting in images with different nuclear density. For tumoral regions, Cellpose was run in 3D, applying the CPx model without retraining with a diameter of 8 μm, mask threshold −0.1, and mask quality threshold 0.3. Objects smaller than 10 μm^2^ in XY area were excluded from the nuclei count. For contralateral and peritumoral regions, Cellpose was run in 3D, applying the Cyto2 model without retraining with a diameter of 10 μm, mask threshold 0, and mask quality threshold 0.3. Objects smaller than 3 μm^2^ in XY area were excluded from the nuclei count. For all regions, objects between 2 and 7.2 μm in Z were counted as single nuclei and objects between 7.2 and 15 μm in the Z dimension were counted as double nuclei to correct for errors in object splitting in Z. Objects smaller than 2 μm in Z were excluded from the analysis to exclude partial nuclei at the edge of the Z‐stack. In all regions, nuclei that were not accurately segmented by Cellpose were added manually. The Cellpose 3D analysis directly provided nuclei per volume.

Single plane confocal images (1 μm thickness) were also obtained from an RG2 tumor rat separate from those studied with MRI/PET. Sections were stained with nuclear stain Hoechst 34580 as indicated above, as well as Iba1 stain (GeneTex [GTX100042]) incubated at 1:400, excited with a 488‐nm laser and CD163 stain (Bio Rad [MCA342R]) incubated at 1:100 excited with a 640‐nm laser.

### 2.3 MRI Procedures

We employed an 11.75 T Bruker Biospec animal MRI system at the OHSU Advanced Imaging Research Center, with a Bruker volume (transmit) coil (72 mm ID) and a homebuilt surface (receive) coil. Rat respiration was monitored and body temperature maintained at 37°C using a warm air temperature control system (SA Instruments). A spin echo DWI sequence was implemented with the following: FOV (20 mm^2^) with outer volume saturation, 1.5 mm slice width, 0.312 × 0.312 mm in‐plane resolution, 10 coronal slices, TR 2.5 s, TE 36.7 ms, diffusion gradient duration/separation 7/25 ms, b values 18, 113, 513, 1013, 2013, 3013, 4513, 6013 [s/(mm)^2^], employing a single diffusion direction (Y). The higher b values were repeated: 2013 (2X), 3013 (4×), 4513 (8×), and 6013 (12×), (acquisition time 80 min). Images obtained from the repeated scans were averaged to increase signal/noise. Post‐contrast T_1_‐weighted images (Omniscan 0.5–1.0 mL *i.p.)* were obtained: FOV 35 × 35 mm, TR 238 ms, FA 60°, 0.091 × 0.091 mm in‐plane resolution, 1.0 mm slice width, 15 slices.

### 2.4 PET Procedures

One day post‐MRI, ^18^F‐FDG PET images were acquired at the Small Animal Research Imaging Core (SARIC) using an Inveon microPET/CT (0.78 × 0.78 × 0.80 mm PET spatial resolution) with the controlling (Inveon Acquisition Workplace [IAW]) and image processing (Inveon Research Workplace [IRW]) software packages (Siemens Healthineers). Rats were fasted by withholding food for 15–18 h prior to PET imaging. ^18^F‐FDG was obtained from Cardinal Health. Dosimetry (1 mCi of ^18^F‐FDG, *i.v*. tail vein) was determined using an Atomlab 500 + Dose Calibrator (Biodex Medical Systems). After a 60‐min latent period between injection and the beginning of PET signal acquisition, a 20‐min PET acquisition was followed by an attenuation CT scan. Image reconstruction of the full 20 min acquisition period was accomplished using an IRW standard preloaded Ordered Subset Expectation Maximization *algorithm*. The CT attenuation correction was applied to raw PET data to produce corrected ^18^FDG‐PET Standardized Uptake Value (SUV) maps. PET data were acquired in Bq and converted to standard uptake value [SUV] using the equation: [SUV = (voxel intensity (Bq/ml)*rat mass (g))/decay adjusted injected activity (Bq)], where the decay‐adjusted injected activity accounts for the decay of ^18^F (half‐life 110 min) over the 60 min latent period between injection and imaging. SUV_max_ for a given ROI was calculated by inserting the maximum voxel intensity in the ROI into the same equation.

### 2.5 MR Image Processing

In‐house MADI software was used to match the voxel DWI signal b‐space decays to an extensive library of decays simulated for various k_io_, V, and ρ combinations, enabling the output of parametric maps of k_io_ (s^−1^), V (pL/cell), ρ (10^5^ cells/μL tissue), vi (intracellular volume fraction), and subsequent calculation of k_io_V and k_io_Vρ. Library parameter bounds were as follows: ρ: 0.044 × 10^5^ – 580 × 10^5^cells/μL; V: 1.13 × 10^−2^ – 2.1 × 10^2^ pL/cell; k_io_: 0–1.3 × 10^2.^ s^−1^; vi: 0.5–0.994. In the library matching analysis, values were not further restricted to defined ranges within these bounds. ADC map creation employed the two point method (ADC = −ln (DW_image_ / T_2image_)*b, with b = 1013 s/mm^2^). PET images were registered to the ADC maps using the JIM 9 image registration tool (Xinapse Systems). Tumor ROIs for both pre‐ and post‐treatment were defined on each imaging slice as appropriate by manually drawing around regions of ADC hypointensity within the right brain hemisphere. Edematous peritumor regions ROIs were defined by manually drawing around regions of ADC hyperintensity proximal to the tumor. Mirror image ROIs were placed in the contralateral regions. In control rats, ROIs were manually defined across the entire brain for neocortex, corpus callosum, striatum, and thalamus, using the Scalable Brain Atlas (Rat—Waxholm Rat) as a guide (https://scalablebrainatlas.incf.org/rat/PLCJB14). The ROIs were transferred to the MADI parameter maps and the co‐registered ^18^FDG PET SUV maps for calculation of mean and median values. Mean and median ROI values were compared with two‐sided paired *t* tests. For histological comparison, MADI ρ ROI medians from the various regions were quantified from the single imaging slice which best matched the histology section examined.

## 3 Results

**FIGURE 2:**
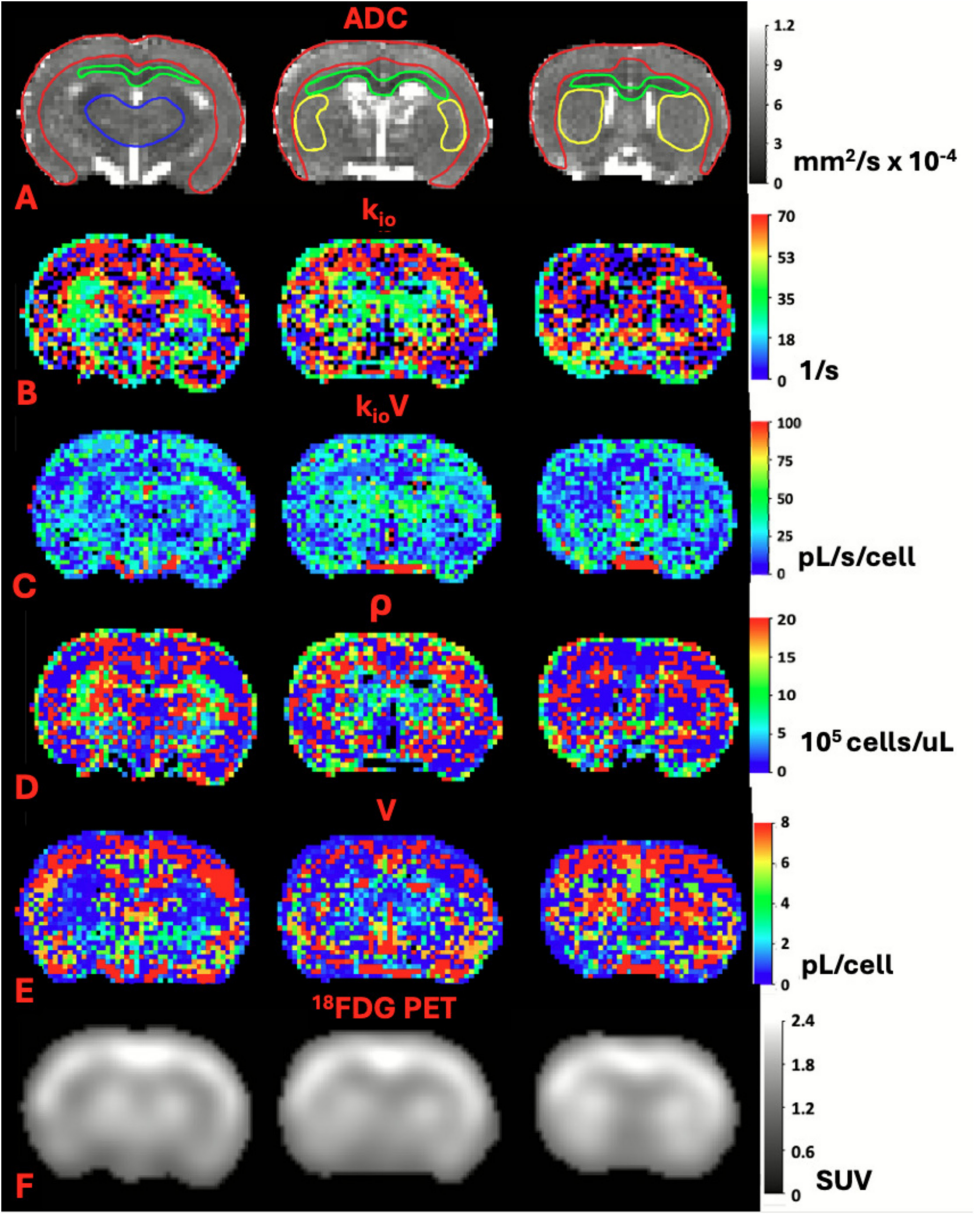
Indicates coronal parameter maps obtained from an untreated non‐tumor bearing control rat, for three contiguous image slice positions through the level of the basal ganglia. (A) ADC, ROIs: cortex: red, corpus callosum: green, striatum: yellow, thalamus: blue; (B) k_io_; (C) k_io_V; (D) ρ; (E) V; (F) ^18^FDG‐PET SUV.

Figure 2 indicates coronal parameter maps obtained from one of the untreated non‐tumor bearing control rats, for three contiguous image slice positions through the level of the basal ganglia. ADC maps are shown in Figure 2A and display ROIs for neocortex (red), corpus callosum (green), striatum (blue), and thalamus (yellow). The means (*n* = 2) of the median voxel values of these ROIs for the measured parameters are shown in Table 1, which also summarizes the means of the ROI median values for untreated tumor rats (*n* = 11) and pre‐ and post‐treated tumor rats (*n* = 5). Various regional differences were observed in the control rats. Figure 2B and Table 1 indicate k_io_ in neocortex to be lower than other regions. The corpus callosum k_io_V (Figure 2C) was lower compared with other regions. The ρ (Figure 2D) for neocortex and striatum was substantially lower than that of thalamus and corpus callosum, while the opposite trend is apparent for V (Figure 2E), as would be expected. Figure 2F indicates that ^18^FDG PET SUV values were relatively high in the cortex, consistent with Table 1 and awake human brain [17, 35].

**TABLE 1:** Indicates medians for all obtained parameters for the indicated regions, for: control rats (*n* = 2), untreated tumor rats (*n* = 11), and treated tumor rats (*n* = 5). The *, **, and *** indicate significant differences from contralateral (*p* < 0.05, < 0.01, < 0.001, respectively, two tailed paired *t* test).

Tissue	ROI volume (mm^3^)	ρ (cell density) (10^5^ cells/μL)	V (cell volume) (pL/cell)	ρV = vi (intracellular fraction)	k_io_ (1/s)	k_io_V (pL/s/cell)	kioVρ (pL/s/𝜇L tissue) ×10^6^	ADC (mm^2^/s) × 10^−4^	SUVmax
Control (*n* = 2)									
Neocortex	323 ± 31	4.02 ± 2.60	3.89 ± 2.7	0.923 ± 0.0263	12.8 ± 9.18	19.7 ± 1.87	8.42 ± 5.9	6.22 ± 0.247	2.73 ± 0.219
Thalamus	56.4 ± 1.9	13.5 ± 2.36	0.667 ± 0.097	0.873 ± 0.0230	43.6 ± 3.96	18.3 ± 0.539	24.6 ± 3.6	5.57 ± 0.0145	2.35 ± 0.221
Corpus callosum	58.8 ± 2.0	17.3 ± 1.69	0.528 ± 0.059	0.922 ± 0.0263	39.6 ± 0.0	13.2 ± 0.737	23.1 ± 3.5	4.41 ± 0.000318	2.58 ± 0.177
Striatum	66.3 ± 5.3	4.09 ± 2.08	3.13 ± 1.8	0.898 ± 0.00272	22.9 ± 10.8	19.6 ± 2.87	8.6 ± 5.3	5.91 ± 0.325	2.59 ± 0.303
un‐Tx (*n* = 11)									
Tumor	29.4 ± 5.3	27.5 ± 4.0 ***	0.46 ± 0.11	0.885 ± 0.006	40.7 ± 2.9	10.5 ± 0.6***	36.0 ± 2.6	4.29 ± 0.19***	7.67 ± 1.50**
Contralateral	29.4 ± 5.3	10.4 ± 1.8	2.00 ± 0.71	0.889 ± 0.009	34.9 ± 5.9	21.7 ± 1.4	30.8 ± 5.2	6.32 ± 0.110	4.16 ± 0.60
Peritumor	54.4 ± 6.5	7.61 ± 0.4*	1.06 ± 0.04	0.835 ± 0.012***	44.3 ± 2.2	42.0 ± 2.6***	37.0 ± 2.0	8.2 ± 0.36*	6.9 ± 1.3**
Peritumor Contralateral	54.4 ± 6.5	9.51 ± 0.9	1.03 ± 0.18	0.888 ± 0.006	38.4 ± 2.4	23.9 ± 1.5	35.0 ± 2.1	6.79 ± 0.29	4.4 ± 0.7
Pre‐Tx (*n* = 5, w/Tx)									
Tumor	19.9 ± 2.2	29.0 ± 6.1	0.380 ± 0.10	0.886 ± 0.0092	38.4 ± 1.2	9.25 ± 0.74**	34.0 ± 1.0	3.87 ± 0.31***	5.98 ± 1.7
contralateral	19.9 ± 2.2	11.4 ± 2.4	1.56 ± 0.91	0.879 ± 0.012	36.2 ± 7.6	20.9 ± 1.9	31.6 ± 6.6	6.09 ± 0.20	3.55 ± 0.76
Post‐Tx (*n* = 5)									
Tumor	14.4 ± 7.0	23.6 ± 7.2	0.76 ± 0.40	0.896 ± 0.0001	34.6 ± 7.1	12.0 ± 1.6*	30.2 ± 6.2	4.60 ± 0.31	4.67 ± 0.73
Contralateral	14.4 ± 7.0	10.0 ± 4.0	1.95 ± 0.86	0.845 ± 0.055	33.1 ± 7.5	20.0 ± 2.3	25.6 ± 5.4	7.68 ± 1.50	4.21 ± 0.34

**FIGURE 3:**
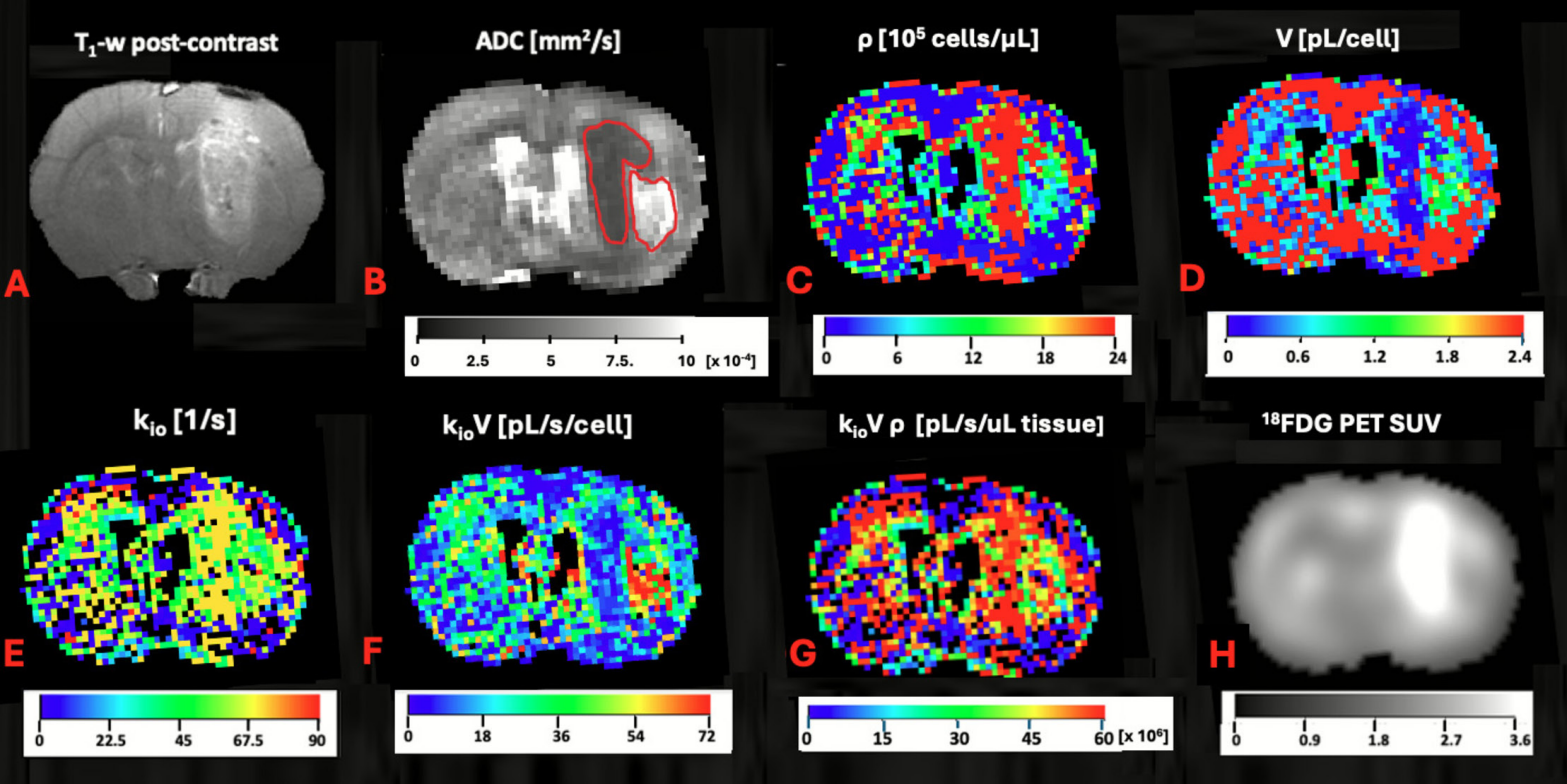
Brain images and parametric maps from a tumor‐bearing rat at equivalent image slice positions: (A) T_1_ weighted post‐contrast image; (B) ADC map, ROIs indicate tumor and peritumoral region; (C) rho ρ map; (D) V map; (E) k_io_ map; (F) k_io_V map; (G) k_io_Vρ map; (H) ^18^FDG PET [SUV] uptake map. Ventricles masked, panels C–G.

Figure 3 displays various brain images and parametric maps at the same slice position from a tumor bearing rat. Figure 3A shows a post‐contrast T_1_‐weighted image with substantial enhancement defining a right hemispheric tumor. Figure 3B displays the ADC map; comparison with Figure 3A reveals that the tumor region is ADC‐hypointense. The ADC map also indicates hyperintense regions peripheral to the tumor; this was a general observation for all rats.

Increased cell density is a hallmark of glioma and solid tumors in general [36]. Consistent with this, Figure 3C,D clearly indicates elevated tumor ρ and decreased V in the tumor region, respectively. Figure 3E,F indicates the MADI parameter maps k_io_ and k_io_V. Consistent with the control rat observations (Figure 2, Table 1), cortical regions tended to have lower k_io_ values than other brain regions. The tumor region tended to have higher k_io_ values than other brain regions. Notably, k_io_V showed a markedly hypointense tumor region, thus indicating a decreased tumor AWC *per cell*. In contrast, the peritumoral region indicated hyperintense k_io_V values. Differing from the tumor k_io_V hypointensity, the relatively increased tumor values of tumor k_io_Vρ (Figure 3G) indicate that *per tissue* AWC is increased. The unique utility of these various combination parameters is discussed in more detail below (see Discussion). Consistent with what has been documented in other ^18^FDG PET studies, the co‐registered ^18^FDG PET image (Figure 3H) clearly indicates a robust tumor tissue^18^FDG uptake [37].

**FIGURE 4:**
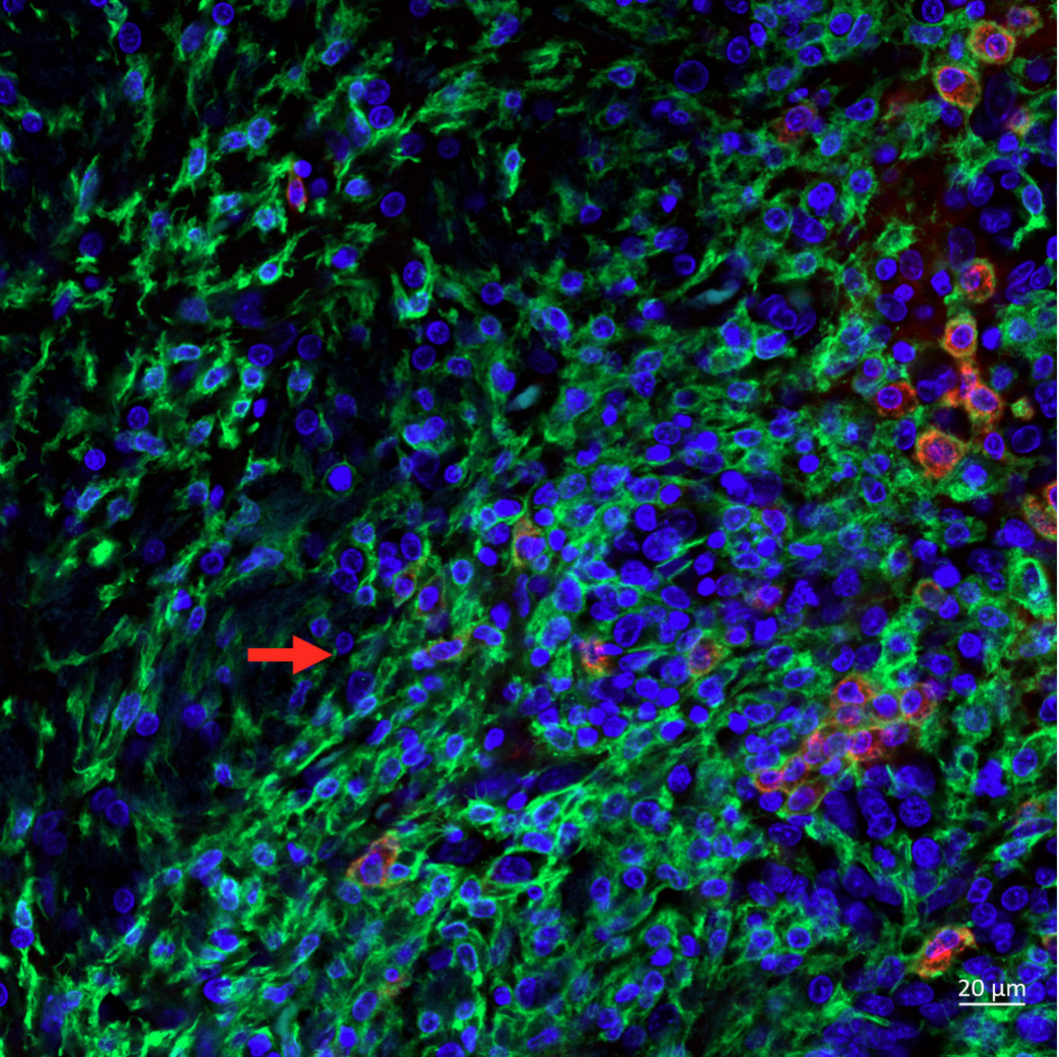
Overlaid confocal images are shown from a histological brain section from a rat implanted with RG2 brain tumor with stains specific for nucleus (Hoechst 34580, blue), Iba1 localized to macrophages and microglia (GeneTex [GTX100042], green), and CD163 localized to M2 macrophages (Bio Rad [MCA342R], red). The tumor border, (see arrow) demarcates tumor (right) and peritumoral regions (left). A higher cellular density is observed in tumor than in peritumor. Notably, the tumor and peritumoral regions indicate numerous macrophages and microglia. While of somewhat lower density in the peritumoral region, a strong peritumoral presence of immune cells and inflammation is clearly indicated. The tumor region also indicates the presence of M2 macrophages.

It has been well documented that non‐contrast enhanced lesion (NCEL) on the tumor periphery as observed in Figure 3, are generally edematous regions indicating hyperintensity on T2/FLAIR and ADC and contain numerous immune cells [38, 39, 40, 41]. Figure 4 indicates overlaid confocal images of histological brain sections obtained from a rat separate from those studied with MRI/PET that was implanted with RG2 brain tumor. The figure shows stains specific for nucleus (blue), Iba1 (green, macrophages and microglia), and CD163 (red, M2 macrophages). The images show a higher cellular density in tumor than in peritumor, consistent with the observation of peritumoral edema in the MRI data. Notably, the tumor and peritumoral regions indicate numerous macrophages and microglia. While of somewhat lower density in the peritumoral region, the images clearly indicate a strong peritumoral presence of immune cells and inflammation. Consistent with the literature, the tumor region also indicates the presence of M2 macrophages, known to be associated with pro‐tumorigenic functions within the tumor microenvironment [42].

**FIGURE 5:**
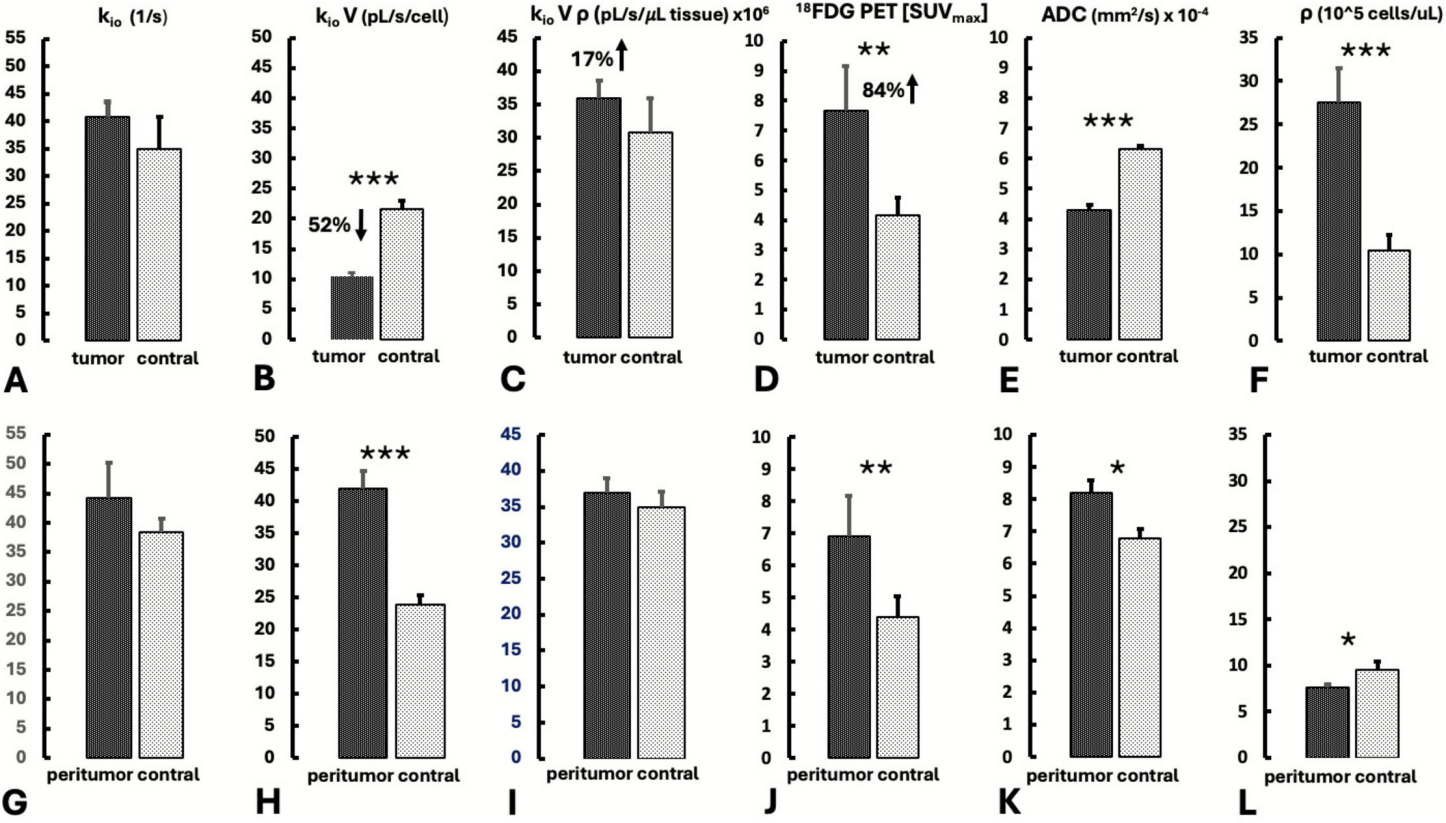
Summarized tumor (A–F) and peritumoral (G–L) data compared to data from their respective contralateral regions. Values indicate the mean (*n* = 11), of the individual median ROI values from each rat (± SE); for k_io_: panels A and G; k_io_V: panels B and H; kioVρ: panels C and I; ^18^FDG PET [SUV_max_]: panels D and J; ADC: panels E and K; ρ: panels F and L. The *, **, and *** indicate significant differences from contralateral (*p* < 0.05, < 0.01, < 0.001, respectively, two tailed paired T test).

Figure 5 indicates summarized tumor and contralateral data and for various MADI metabolic and cytometric parameters as well as ADC and ^18^FDG PET. To mitigate the influence of voxel outlier values, the mean of the individual median (*n* = 11) ROI values from each rat are reported. Figure 5A indicates that tumor k_io_ trended slightly higher than contralateral k_io_ but was not significantly different. In contrast, k_io_V in Figure 5B, clearly indicates a mean value that is less than half of that of contralateral (*p* < 0.001), consistent with Figure 3. Notably, this pattern differs from that observed in Figure 5D, which indicates that tumor ^18^FDG uptake was approximately double that of contralateral (84% increase, *p* < 0.01), as is generally observed [37]. Because k_io_V quantifies water efflux *per cell*, the substantially reduced tumor V in comparison with contralateral (Table 1) affects the relative k_io_V values. In contrast, the ^18^FDG PET quantifies glucose uptake on a *per tissue* basis and is independent of V. However, direct comparison to^18^FDG PET SUV_max_ is facilitated by the *per tissue* transcellular water transport efflux rate k_io_Vρ (Figure 5C), which in contrast to k_io_V indicated slightly higher values for tumor (17%, NS). The comparison of MADI parameters and ^18^FDG PET SUV_max_ values provides information regarding oxidative versus glycolytic energy production and is further discussed below (see Discussion). Tracking the k_io_V pattern, the ADC (Figure 5E), indicates a substantially lower tumor ADC versus contralateral. Figure 5F indicates that the tumor ρ is greatly increased versus contralateral, consistent with Figure 3C. Data from the peritumoral regions, are shown in the bottom row panels. Figure 5G indicates that k_io_ for peritumoral trended slightly higher than its contralateral region (NS). Notably, Figure 5H indicates that, in contrast to tumor, the peritumoral k_io_V is substantially higher than its contralateral region (*p* < 0.001). The similar V values (Table 1) in these regions excludes V as the origin of these *per cell* differences, hence suggesting different metabolic phenotypes, with increased AWC and energy turnover in peritumoral versus contralateral, possibly a characteristic of the inflammatory immune cells, which largely constitute the peritumoral regions. In contrast, the analogous *per tissue* parameter, k_io_Vρ, (Figure 5I) indicates similar values for peritumoral and contralateral, likely related to the increased edema, which reduces peritumoral *per tissue* values, consistent with the significantly lower ρ and intracellular volume fraction, vi, (Figure 5L and Table 1, respectively). Figure 5J indicates the peritumoral ^18^FDG PET SUV_max_ was increased over contralateral, which may be related to the known glycolytic energy dependence of the aforementioned peritumoral cell types [43]. However, partial volume effects from the tumor may also contribute to this, due to the lower resolution of PET versus MRI. Analogous to observations with the tumor, the peritumoral ADC (Figure 5K) tracked the k_io_V, indicating higher values than the contralateral.

**FIGURE 6:**
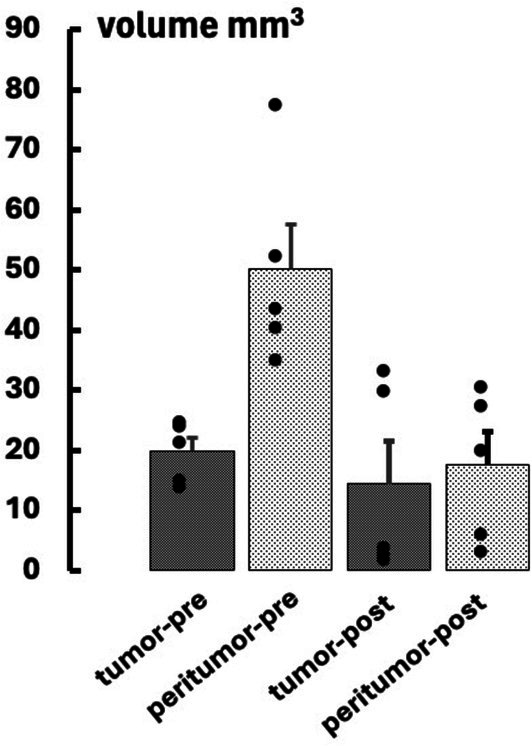
Volumes of tumor and peritumoral regions are shown at pre‐ and post‐treatment.

Figure 6 indicates mean pre‐ and post‐treatment tumor and peritumor volumes for the (*n* = 5) rats that received CRT. In the 5–6 days post‐treatment, three of the five treated tumors substantially decreased in volume while two moderately increased; tumor volume did not significantly change while peritumoral volume indicated a strong tendency to decrease (*p* = 0.056).

**FIGURE 7:**
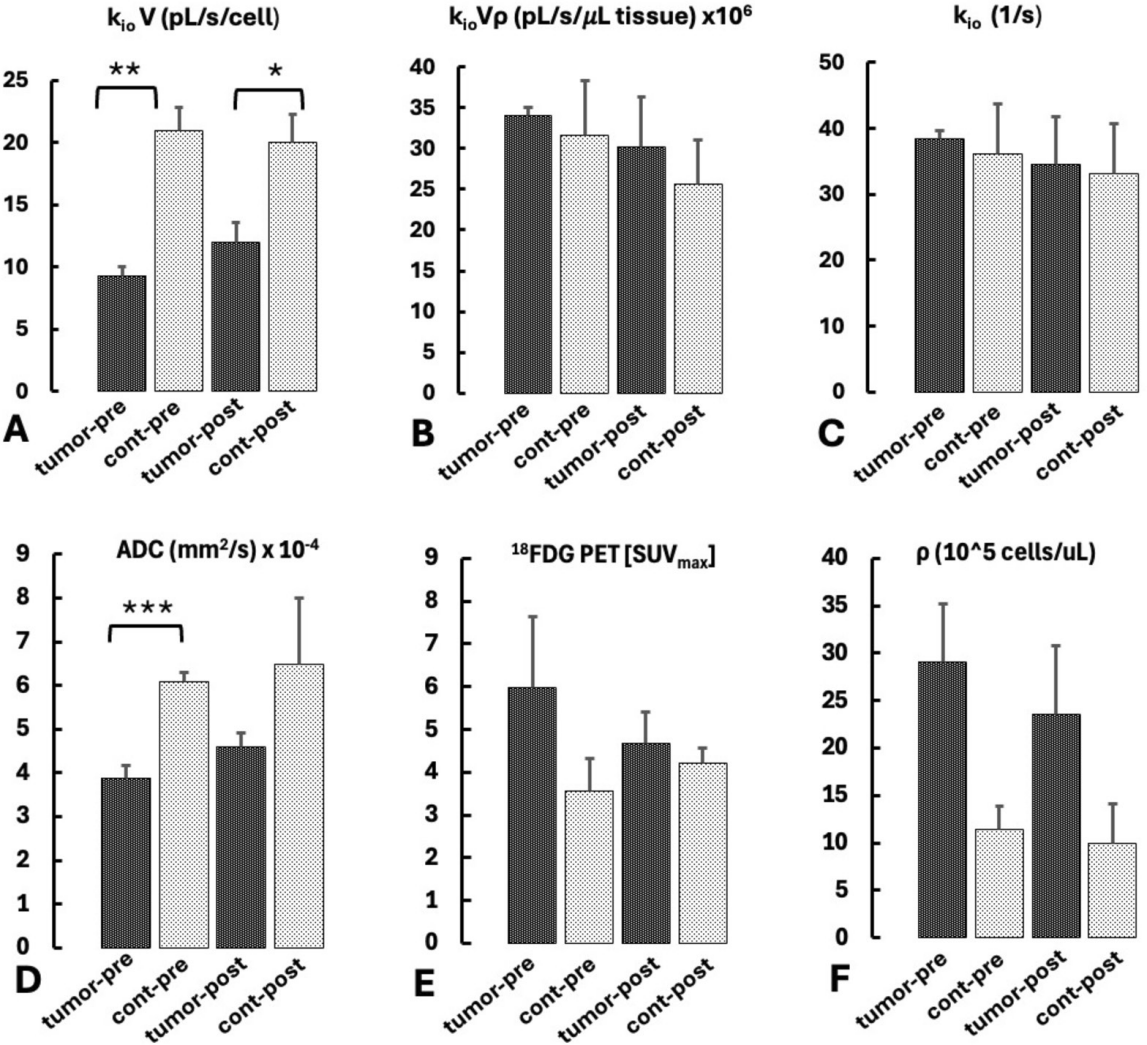
Summarized tumor data from (*n* = 5) treated rats both before and after treatment, compared to their respective contralateral regions. Values indicate the mean of the individual median ROI values from each rat (± SE); for (A) k_io_V; (B) k_io_V ρ; (C) k_io_; (D) ADC; (E) ^18^FDG PET [SUVmax]; and (F) ρ. The *, **, and *** indicate significant differences from contralateral (*p* < 0.05, < 0.01, < 0.001, respectively, two tailed paired T test).

Figure 7 indicates summarized pre‐ and post‐treatment MRI and PET data. Consistent with Figure 5, pretreatment k_io_V (Figure 7A) tumor values were significantly lower (*p* = 0.005) than contralateral while ^18^FDG PET [SUV_max_] (Figure 7E) tended to be higher (*p* = 0.062). The post‐treatment k_io_V pattern did not differ substantially from pretreatment, with post‐treatment tumor k_io_V remaining significantly different from contralateral and not different from pre‐treatment. Similarly, the tumor k_io_Vρ (Figure 7B) and k_io_ (Figure 7C) did not show significant differences between pre‐ and post‐treatment. The tumor ^18^FDG PET [SUV_max_] tended to decrease post‐treatment (NS), becoming similar to contralateral, but variability precluded definitive conclusions regarding detection of treatment response. Analogous to k_io_V, the pre‐treatment tumor ADC (Figure 7D) showed significantly lower values than contralateral, and increased only marginally at post‐treatment. Analogous to Figure 5, the pre‐treatment ρ (Figure 7F) tended to be greater for tumor than contralateral (*p* = 0.055); treatment did not substantially reduce its value (NS).

**FIGURE 8:**
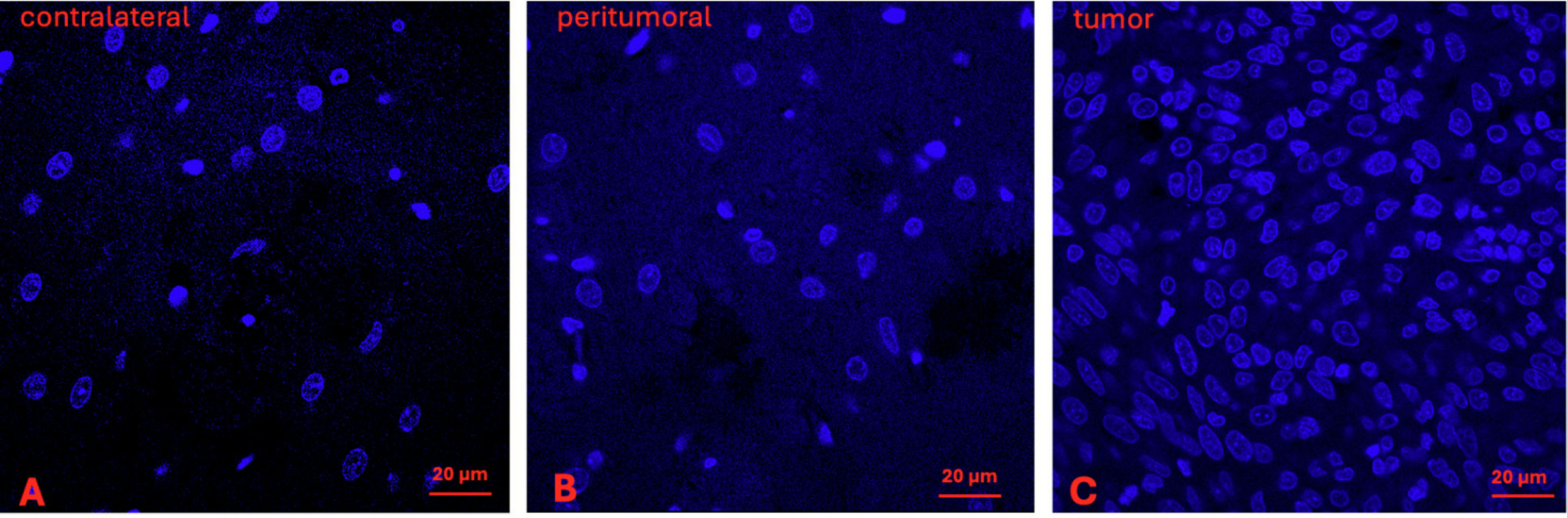
Representative single plane image slices from confocal Zstacks obtained from an untreated rat brain histological section stained with the fluorescent nuclear stain Hoechst 34580, for contralateral (A), peritumoral (B) and tumor (C) regions, respectively.

**FIGURE 9:**
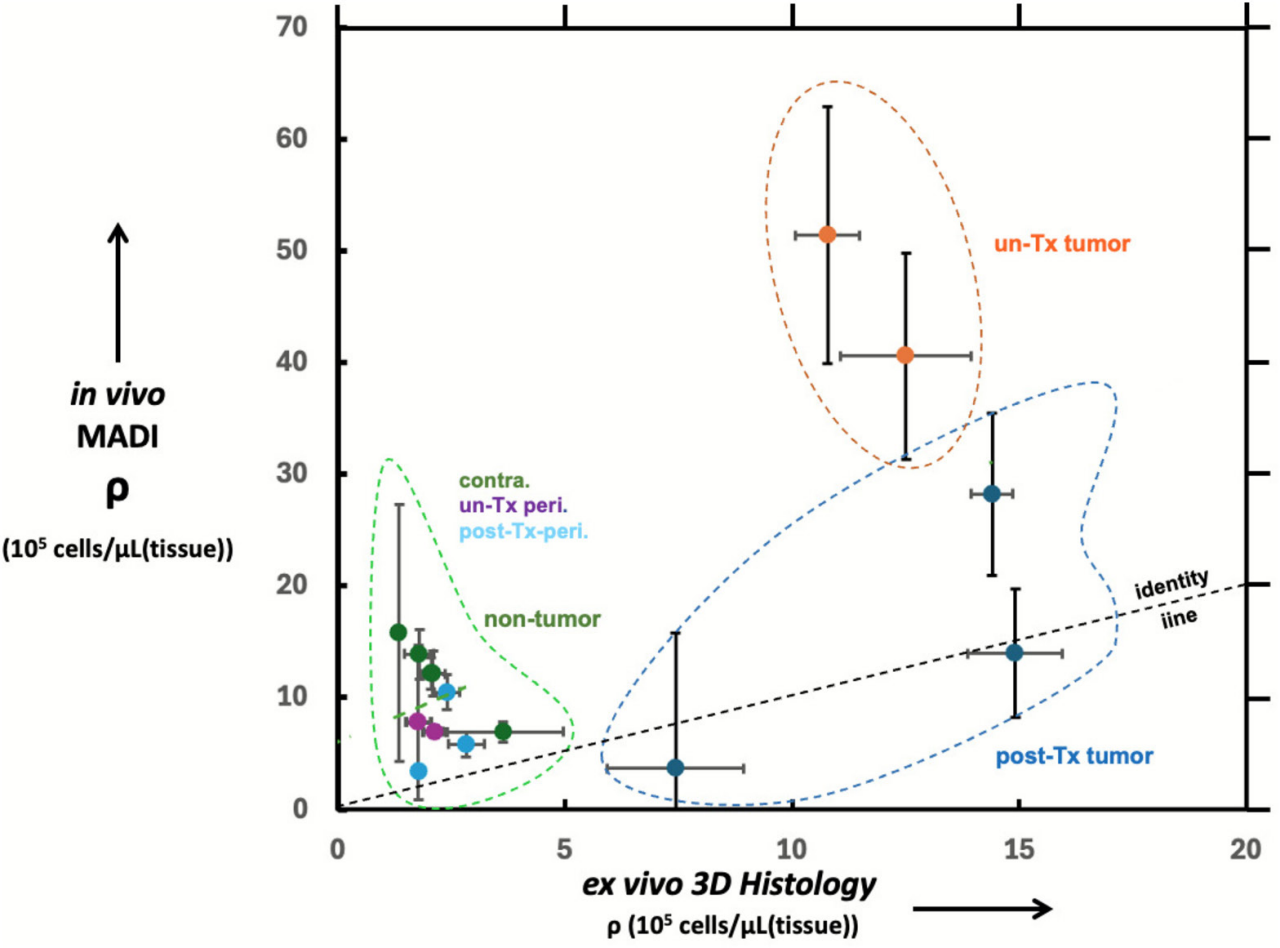
MADI in vivo ρ values from individual rats are plotted against those obtained from a 3D histological analysis of coronal tissue sections from several [5] of the rats, for untreated and post‐treatment tumor, untreated and post‐treatment peritumor, and combined (untreated and post‐treated tumor) contralateral regions. The MADI ρ values are medians of ROIs of the indicated regions taken from a single imaging slice which best matched the histology section examined (±SE). Histology ρ data are means (±SE) of the 3 visual fields assessed within the targeted regions. The dashed line represents a line of identity.

Figure 8 shows single plane images from example confocal Zstacks obtained from an untreated rat brain histological section for contralateral (Figure 8A), peritumoral (Figure 8B) and tumor regions (Figure 8C), respectively. The panels show that the tumor region has substantially greater nuclear density than contralateral and peritumoral regions. A 3D histological analysis of coronal tissue sections from several of the rats directly provided ρ values that were compared to the in vivo MADI values (Figure 9). Median MADI ρ values from the ROIs (tumor, contralateral, peritumor) as defined above (see Experimental section), were taken from a single ρ parameter map slice which best corresponded with the histological section studied. The MADI ρ values from two pretreatment tumors and three post‐treatment tumors for the various regions (tumor, peritumor, contralateral) were plotted against the values obtained histologically from the analogous regions. Contralateral included both treated and untreated (*n* = 5). The histological ρ values plotted were mean values from 3 Zstack visual fields analyzed within a region of an individual brain. Shrinkage of histological sections was estimated and accounted for by comparing whole brain slides with the corresponding in vivo MR images and found to be a 3% linear shrinkage in the coronal plane, corresponding to a volume shrinkage of 9%. A line of identity is shown to facilitate comparison between the MADI and histological values, which is further discussed below (see Discussion).

## 4 Discussion

**FIGURE 10:**
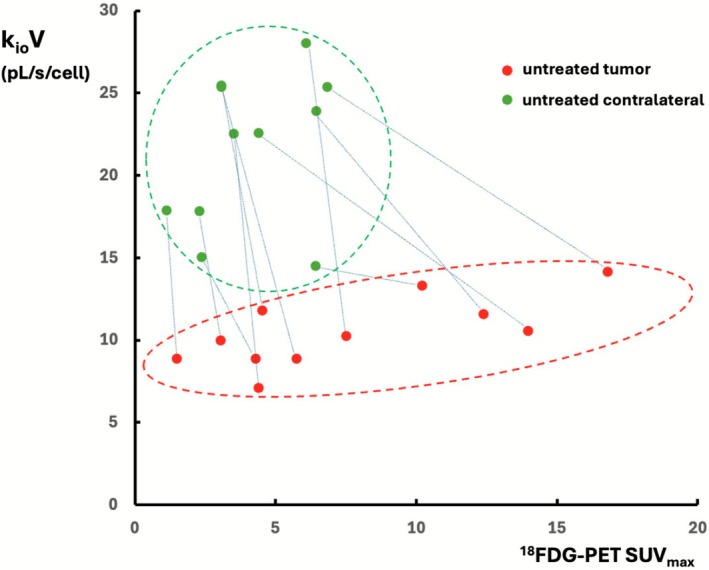
Individual rats ROI k_io_V medians for the (*n* = 11) untreated rats, are plotted against ^18^FDG PET [SUV_max_]. Connector lines are placed between tumor‐contralateral pairs.

While MRI and computed tomography (CT) provide exquisite high‐resolution anatomical imaging, metabolic imaging is the next frontier for improving disease assessment and diagnosis. There is increasing interest in mapping tumor k_io_ values as a biomarker of cellular water efflux [9, 10, 11, 12, 13], which has been found to correlate with tumor aggressiveness in rodent models [9, 12]. Recent reports correlated k_io_ with aquaporin‐4 expression and activity as well as proliferation in glioma cell culture, preclinical glioma models, and human glioma [10, 13]. Changes in k_io_ have also been correlated with tumor treatment in a murine glioblastoma model [11]. Such experiments demonstrate the utility of water efflux measurements as a metabolic biomarker in studies of cancer and cancer treatment. To date, such studies have employed some variant of DCE‐MRI data analyzed using the shutter‐speed paradigm (SSP) [27, 44]. This approach has two key limitations which MADI addresses. First, the SSP approach requires an extracellular contrast agent, and it has to be concentrated enough to increase the shutter‐speed sufficiently [17]. This precludes studies in brain with an intact blood–brain barrier and, even in brain tumors, which have a compromised BBB, the concentration may not reach sufficient levels for accurate k_io_ determination [17]. The second limitation is that while SSP can determine the intracellular water fraction, vi, it cannot separately determine the two vi components, V and ρ (vi = Vρ product). In contrast, MADI can quantify V and ρ. Various other DWI‐based approaches have been developed to measure cell size in tissues, generally using combined PGSE and OGSE DWI scan protocols and an assumed spherical cell geometry [45, 46, 47, 48]. Notably, however, these do not provide measurements of water efflux measurements. MADI also uses a more sophisticated Voronoi cell structure model instead of spherical to accommodate a greater variety of cell shapes [18]. MADI's cytometric and metabolic parameters can also be combined to provide other important parameters, that is, k_io_V and k_io_Vρ. With the rat glioma model employed in the current study, k_io_V demonstrated a greater ability than k_io_ to discriminate tumor from contralateral in the rat glioma model employed; significant differences were observed between untreated tumor and contralateral for k_io_V and ρ, but not for k_io_ (Table 1, Figure 5). While both k_io_V and ^18^FDG‐PET SUV_max_ indicated significantly different values for tumor versus contralateral (Figure 5, Table 1), advantages of k_io_V over SUV_max_ in tumor discrimination are shown in Figure 10, which plots data points for individual rats ROI medians, for the (*n* = 11) untreated rats. Connector lines between untreated tumor‐contralateral pairs demonstrate that although ^18^FDG‐PET SUV_max_ was consistently greater than its associated contralateral, differences were minimal in some rats, and overall, substantial overlap was observed in the absolute values of tumor and contralateral ^18^FDG‐PET SUV_max_ (x axis). This variability of ^18^FDG‐PET uptake in brain is a well‐known limitation affecting tumor evaluation [37]. In contrast, there is essentially no overlap between the k_io_V values (y axis) of untreated tumor and those of contralateral. Further k_io_V utility was demonstrated by its ability to discriminate tumor and peritumoral regions. Glioma peritumoral areas are generally inflammatory, edematous regions containing both normal cells and invading tumor cells [38, 39, 40, 41]. Consistent with edema, the ρ and vi parameters provided by the MADI analysis showed a clear reduction compared with contralateral (Figure 5, Table 1). Notably, in contrast to the lower k_io_V values indicated by the tumor, the peritumor indicated much higher k_io_V values than contralateral, suggesting the peritumoral region to be metabolically distinct from the tumor region. Notably, this distinction was not evident in the ^18^FDG‐PET images, which showed elevated ^18^FDG uptake in both regions. The definition k_io_V provides between tumor and peritumor may provide a key advantage in tumor treatment assessment, particularly with the crucial issue of distinguishing tumor recurrence from treatment‐related changes (TRC) such as pseudoprogression (PSP) and radiation necrosis (RN) [49, 50, 51]. TRC is associated with inflammation and permeability changes induced by radiation and/or chemotherapy and can induce contrast enhancing effects and perilesional edema, which can appear similar to recurrent tumor with standard MRI approaches. ^18^FDG PET has been shown to modestly improve distinction between TRC and recurrence [50, 51]. However, ^18^FDG has significant uptake in normal brain tissue, hence reducing the tumor‐to‐background ratio and can also exhibit increased uptake in inflammatory regions. Various amino acid PET tracers have been shown to provide improved TRC/recurrence distinction, such as 6‐fluoro‐(^18^F)‐L‐3,4‐dihydroxyphenylalanine (FDOPA), L‐[Methyl‐^11^C]‐Methionine (MET), ^18^F‐fluoroethyl‐tyrosine (FET), and ^18^F‐fluciclovine (Axumin) [36, 37, 43]. However, of these, only FDOPA and Axumin are FDA approved in the USA, with the other two requiring an Investigational New Drug application to use, and MET has such a short half‐life that it requires an on‐site cyclotron. While some studies have shown ADC parameter maps to assist in distinguishing glioma recurrence from TRC, there have been conflicting reports as to the direction of the comparative ADC changes [50]. In the current study, ADC maps demonstrated clear tumor demarcation (Figures 3 and 5). ADC also generally tracked k_io_V in terms of the observed trends. It is important to note, however, that while in this model the directional changes were similar for ADC and k_io_V, they constitute very different measures. ADC is b‐dependent, provides only a general quantification of diffusion, and is affected by various cytometric and metabolic considerations. In contrast, the MADI approach deconvolves the DWI information into its underlying factors, which provide key information regarding both cell structure and metabolism, with k_io_V specifically quantifying the rate of water efflux per cell. It is thus a unique parameter, which is likely to be useful in studies of tissue metabolism. MADI also provides an advantage over ^18^FDG‐PET and contrast‐based approaches for tumor assessment in that it does not require a contrast agent or radioactive tracers, and has greater resolution than ^18^FDG‐PET. We hypothesize that the ability of MADI to simultaneously provide metabolic and cytometric information is likely to provide clinical value for tumor assessment and TRC/recurrence distinction. As a key focus of this study was to compare MADI metabolic metrics to those from the most common metabolic imaging approach, ^18^FDG‐PET, it was beyond the scope of this study to compare MADI to other non‐metabolic MRI cancer assessment strategies (i.e.*,* DSC, DCE, etc.). Future studies are required to assess MADI in that context. The primary strength of MADI is its provision of metabolic and cytometric quantities, which we believe will have potential utility in disease assessment.

Our measurement of MADI parameters in the various brain regions of the control rats indicated values in the same range as recently reported in our recent human brain MADI study as well as similar patterns distinguishing the various brain regions, thus indicating a consistency both in the MADI approach and between mouse and human [17].

Our observation of lower ρ in neocortex versus other brain regions is consistent with our human MADI work and with isotropic fractionation results in rat brain [52]. As with the human study, the current study measured lower k_io_ values in cortex and striatum compared to thalamus and corpus callosum. A substantially lower k_io_V in corpus callosum than in the other assessed regions was also observed in both studies. Also analogous to our human work and those of others, the current study found both k_io_V and ^18^FDG PET SUV_max_ to be highest in the cortex compared with the other regions [17, 35].

The current study represents the first to compare MADI ρ values obtained in vivo with histological ρ values obtained from the same tissue. We utilized a 3D histological approach, using a Zstack of confocal microscopy images to count nuclei visualized with a fluorescent nuclear stain within a 20‐μm slice to ensure that they were fully counted within a designated tissue space. This approach provides cells‐per‐volume directly rather than the cells‐per‐area provided by a 2D approach with a single image and limited focal depth. Our study employed a Cellpose algorithm to segment and count the nuclei, which enabled complete visualization of the entire confocal Zstack as well as what nuclei were being counted. Missed nuclei were manually added. Filters excluded objects too small to be nuclei or partial nuclei at the edge of the Z stack, and close nuclei that were not accurately separated by the algorithm were accounted for as described above. Figure 9 compares the ρ values obtained from MADI in individual animals with that from the histological analysis. Comparison of in vivo measurements with histological analysis of preserved tissue always presents challenges. While the MADI map slice was chosen to roughly match the histological section employed, the ROI includes the entire targeted region while the histology averages data from three relatively small fields within the region, and regional heterogeneity can add uncertainty to the comparison. The known heterogeneity of tumors likely contributes to the increased scatter of the tumor data compared to the contralateral and peritumor data, which tended to be clustered at lower ρ values. This was particularly true for untreated tumor p compared to post‐treated; however, definitive conclusions regarding the comparison of the two tumor groups were limited by sample size limitations and the considerations noted above. Across the range of values, the majority of data points are above the line of identity, indicating a higher MADI ρ than histology ρ. Consistent with this, linear regression through all points indicates a slope and intercept of 1.73 and 6.1, respectively (*R* ^2^ 0.40, *p* = 0.011). A possible explanation is a method‐related bias in MADI ρ calculation. Various experimental details and observations argue against a substantial underreporting ρ values by histology; shrinkage was taken into account, the nuclear stain employed is known to stain nuclei from all cells, and notably, the confocal Zstacks from tumor regions (Figure 4, Figure 8C) generally indicated a very high nuclei density, which could not have accommodated a substantially higher density of cells. We considered the possibility that MADI is detecting structures that histology did not. While the 3D confocal approach minimizes the undercounting of nuclei, in non‐tumor (i.e.*,* contralateral) regions a small degree of histological undercounting of nuclei could theoretically occur with neuropil structures such as axons and dendrites due to their length. In tumor, the high cell density precludes additional structures except those of a relatively small size. Such structures might include, for example, apoptotic bodies (ApoBDs), which are small (0.5–2 μm diameter) compared with cancer or immune cells (10–20 μm diameter) and can be present in tumors [53]. They often contain fragmented nuclear material, which stains very brightly and are generally quickly cleared by macrophages shown to be prevalent in the RG2 tumor model (Figure 4) [54]. The confocal analysis did not indicate evidence of such structures. Each Zstack was carefully examined throughout in the process of manually targeting nuclei that were missed by the software. Our general observation was that small stained objects were very few in number compared to the number of tumor nuclei. If observed, they were generally at the edges of the Zstack, representing partial nuclei and were filtered out by the Cellpose software. Consistent with this, the apoptotic index (apoptotic cells divided by total cells) is generally < 1% in glioblastoma tumors [55, 56]. This in combination with their relatively small size suggests that apoptotic bodies would constitute an insignificant fraction of the voxel‐averaged signal and they would not substantially contribute to the MADI ρ value; smaller extracellular vesicles would constitute an even lower contribution. Taken together, while a contribution of smaller structures to MADI ρ that are histologically undetected cannot be entirely excluded, our current evidence suggests that this does not account for the differing ρ values. However, the moderate but significant correlation observed between MADI and histology ρ values provides validation for the concept that MADI is sensitive to and tracks ρ. A greater sample size may be required to better characterize the precision and accuracy of MADI, particularly in heterogeneous tumors for which histology registration to MADI parameter map ROIs can be challenging. A more accurate characterization might result from testing MADI in a less complex, more uniform model system such as samples of packed cells of relatively uniform size (e.g., red blood cells or yeast cells).

While MADI and ^18^FDG‐PET assess different metabolic processes, they provide complementary information which together can provide an informative assessment of tumor energy metabolism. Unique to the MADI approach, the k_io_V and k_io_Vρ combination parameters quantify the rate of AWC *per cell* (^c^MR_AWC_) and *per tissue* (^t^MR_AWC_), respectively. In contrast, ^18^FDG‐PET provides a measure of glucose uptake, and as with previous studies, we observed greatly increased tumor ^18^FDG uptake compared to contralateral brain, consistent with the upregulated tumor glycolytic energy production associated with the Warburg Effect. Employing the conversion factor obtained from the linear calibration plot in a mouse ^18^FDG‐PET study [30], SUV_max_ can be directly converted to the rate of tissue glucose uptake, ^t^MR_glc_: 3.2*[SUV_max_] = [pmole (glc)/s/μL (tissue)]. Division of this quantity by the MADI parameter ρ further converts this to a per‐cell quantity ^c^MR_glc_ = [pmole (glc)/s/cell]. MADI's ability to determine V and ρ distinguishes it from PET and MRSI quantities, i.e., per unit (volume or mass) tissue, and so enables the ability to determine whether a change in a *per‐tissue* quantity is due to a change in *per‐cell* quantity, or due to ρ, or both [16]. The *per‐tissue* tumor SUV_max_ quantity was significantly higher (84%) than contralateral (Figure 5, Table 1), as were the derived values of ^t^MR_glc_ for tumor and contralateral (24.6 ± 4.8 and 13.3 ± 1.9 pmole (glc)/s/μL (tissue)). In contrast, the analogous *per‐cell* quantity ^c^MR_glc_ indicated a reverse trend, with lower levels observed in tumor (15.7 ± 7.8 and 22.9 ± 8.2 amole (glc)/s/cell for tumor and contralateral, respectively (NS)). The divergent patterns derive from the higher ρ (and smaller V) for tumor versus contralateral in this RG2 tumor model. Hence the increased *per‐tissue* glucose uptake is primarily due to a higher ρ and is not due to increased glucose transport on a *per‐cell* basis.

The often‐observed upregulation of tumor tissue glucose uptake has led to a common perception that energy requirements are increased in tumors, ostensibly related to the increased cell proliferation. However, the increased glucose uptake is primarily related to a (partial) switch that occurs in tumors, from oxidative to glycolytic energy production. Glycolytic energy production produces only 2 ATP per glucose, while oxidative produces 36, and so to provide sufficient energy glucose uptake is greatly increased. This switch occurs to increase utilization of the TCA cycle for provision of biosynthetic intermediates needed for cell division and growth [57]. While oxidative energy production is still employed to a substantial degree [58], its reduction requires large amounts of glucose to be taken up due to the relative inefficiency of glycolytic energy production. Hence, the large increase in glucose uptake is not necessarily indicative of an increase in total energy needs of the tumor cell, as reported by a recent study, which measured the rate of total ATP synthesis in healthy and cancerous mouse tissues, from both oxidative phosphorylation and glycolysis [30]. The study did not include gliomas, but indicated that in various solid primary tumor types, total ATP production was actually reduced compared with the analogous healthy tissue; for example, reductions of 80% and 90% in pancreas and colon cancer, respectively, despite the presence of increased glycolysis. The study further documented that such tumors downregulate ATP‐expensive functions characteristic of the tissue of origin, such as enzyme secretion and fat digestion (pancreas), gluconeogenesis, and glycogen metabolism (liver) and bicarbonate and sodium recovery from blood filtrate (kidney). In contrast however, in metastatic tumors, total energy production was increased; the current study suggests this to also be the case in the RG2 rat glioblastoma model. As the k_io_V and k_io_Vρ water efflux rate quantities are sensitive to NKA flux, the primary cellular energy consumer, they also reflect overall cellular energy use. The *per‐cell* k_io_V quantity (Figure 5B) clearly decreases in tumor versus contralateral, a pattern influenced by the disparity in cell volume between the different regions. However, the k_io_Vρ quantity can be employed to compare different regions on a *per‐tissue* basis irrespective of different cell volumes; notably, its value increases by 17% (NS, Figure 5C) in tumor compared with contralateral, consistent with a moderate increase in overall cellular energy turnover. An increased requirement for total cellular energy in the syngeneic rat RG2 glioma would be consistent with reports of increased activity of a number of transcytolemmal Na^+^ transport mechanisms in glioma, many of which transport water as well (Figure 1). As mentioned earlier, the maintenance of ion gradients by the NKA accounts for a large percentage of energy use in the brain. Such increased Na^+^ influx would require NKA upregulation and increased ATP consumption. The moderately increased tissue AWC in tumor detected by MADI suggests increased ion transport and energy consumption. Upregulated Na^+^ transport mechanisms in tumors reportedly include the Na^+^/H^+^ exchanger (NHE1), Na^+^/glucose cotransporter (SLGL2), Na^+^/K^+^/2Cl^−^ co‐transporter (NKCC), acid‐sensing ion channels (ASICs), amiloride‐sensitive epithelial Na^+^ channel (ENaC), N‐methyl D aspartate receptor (NMDA‐R), and various Na‐coupled amino acid and glucose transporters [59]. Their increased activity supports various cancer related functions, including proliferation, migration, invasion, acid extrusion, glutamate and glucose uptake. Consistent with an increased Na^+^ influx in tumors, tissue Na^+^ levels in human glioma tissue has been reported to be substantially higher than in normal brain [2].

**FIGURE 11:**
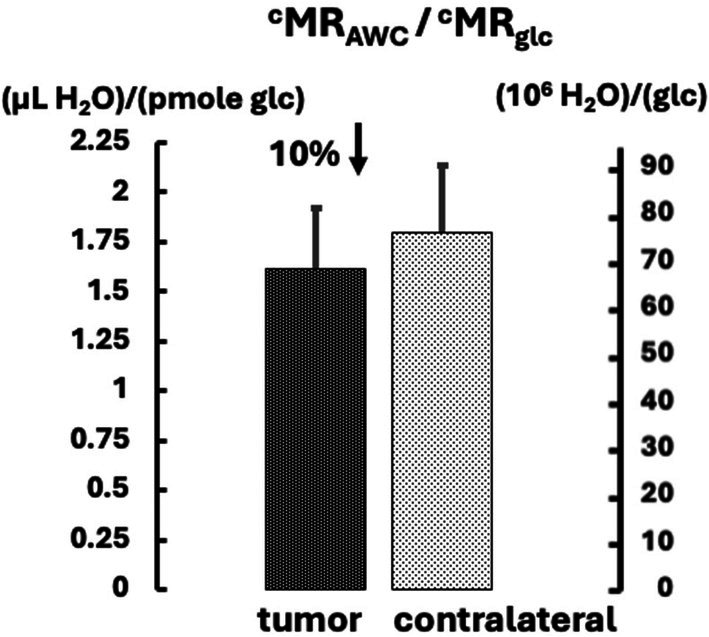
The ratio ^c^MR_AWC_/^c^MR_glc_ for both tumor and contralateral is shown, indicated in units of both μL H_2_0/pmole glucose and in molar ratios of H_2_O/glucose.

The concept that glycolysis is greatly activated in tumors without substantially increasing total energy turnover and also remaining the lesser contributor to it is supported by comparisons between the MADI and PET data. The relatively moderate increase in k_io_Vρ (17%) suggests that the marked ^18^FDG‐PET SUV_max_ increase (84%) was not associated with a large increase in total energy turnover. That glycolysis remains a relatively minor component of total energy production in tumors is also supported by further comparisons. As indicated above, SUV_max_ can be converted to ^c^MR_glc_ and k_io_V defined as ^c^MR_AWC_. The ratio of these quantities constitutes a water glucose index (WGI) and is shown in Figure 11, both in units of μL H_2_O/pmole glucose and in molar ratios of H_2_O/glucose. The comparative decrease in tumors was of small magnitude (10%, NS). Because, as stated above, glycolysis is a very inefficient way to produce ATP in terms of glucose molecules consumed, this is consistent with the concept that the decrease in overall energy production per glucose was marginal. Hence, despite glycolytic activation in tumors, it did not dominate energy production, which would have lowered WGI more substantially. Hence, the MADI approach, in combination with ^18^FDG‐PET, provides a more complete picture of alterations in energy metabolism that occur in cancer cells than ^18^FDG‐PET alone provides.

The k_io_ and k_io_V parameters are different types of metabolic quantities (rate constant versus rate, respectively) and indicated different patterns with respect to how they changed with the various tissue regions. In the tumor model employed, k_io_ clearly was not as sensitive as k_io_V to differences between tumor and contralateral. As cell volume (V) is incorporated into the *per cell* quantity k_io_V, the small tumor cell volume contributes to the decreased k_io_V relative to contralateral. However, while V clearly affects the k_io_V value, it should also be noted that k_io_ is not independent of cell volume; k_io_ is the inverse of the cellular water lifetime τ and tends to increase with decreased cell volume due to surface area to volume considerations. In contrast to k_io_V, k_io_ tended to be somewhat higher in tumor than contralateral. Importantly, a comparison of k_io_ between tumor and contralateral has not been previously accomplished in brain, as with contrast methodology it is not possible to measure k_io_ in normal brain tissue, which is one of the advantages of MADI over contrast‐based methods. Hence, prior to the current study, there was no solid information to guide expectations of tumor/contralateral relative values. The moderate k_io_ differences observed between tumor and contralateral do not diminish the utility of k_io_ as a key metabolic measure. The current study also highlights k_io_Vρ as a particularly useful metabolic quantity. As a *per tissue* quantity, it is more directly comparable to SUV_max_ than the *per cell* quantity k_io_V and is similarly more appropriate for comparison to tissue ion channel activities and overall tissue energy consumption. As was indicated above, its tendency to moderately increase in tumor over contralateral is consistent with reports of increased ion transport activity in glioma (and associated energy consumption to support it). Notably, k_io_ showed the same moderate increase (17%) as k_io_Vρ in tumor over contralateral. Because the Vρ term is equivalent to vi, k_io_Vρ = k_io_ (vi) product. As the vi in tumor and contralateral was quite similar (both were 0.89, Table 1), the k_io_ and k_io_Vρ values would be expected to track each other in these two tissue regions. More studies are required to further assess the utility of these various MADI metabolic parameters. The k_io_V quantifies AWC at the *per cell* level, a unique capability of the MADI approach; the current study suggests its utility for detection of tumor and peritumoral regions. The k_io_Vρ quantity and the related parameter k_io_ are useful for metabolic considerations at the tissue level, making them comparable to other tissue activity measurements.

Treatment effectively reduced tumor volume in several of the tumors while others increased. As a group, treatment changes in the tumor MADI parameters, ADC, or ^18^FDG‐PET SUV_max_ were not detected in the subset of tumors receiving CRT. Sample size limitations prevented meaningful evaluation of responder versus non‐responder tumors, highlighting a potential direction for future studies.

The current study contained certain limitations. MADI requires obtaining DWI data with relatively high b values which have low signal/noise ratios and exacerbates motion artifacts from physiological motion (breathing, arterial pulsation), a particular issue with small animal imaging. This required obtaining multiple averages of images with high b values which lengthened the scan time. Obtaining quality echo planar images (EPI) was not feasible due to equipment limitations of our MRI system at the time of the experiments. We have acquired diffusion tensor trace‐averaging (sequential, orthogonal diffusion directions) normal [17] and tumor‐bearing human brain data of suitable quality for MADI analysis in 10 min on clinical scanners using EPI approaches. In the current study, we reduced motion artifacts by restricting the diffusion direction to one direction (Y). In a non‐tumor bearing rat we sequentially compared this approach with using equally weighted X, Y, and Z diffusion directions, otherwise using the same parameters as employed in the current study. Median ADC and MADI parameter values were quantified in the same [4] brain regions as shown in Figure 2. The percent change in parameters observed when changing from one diffusion direction to three directions was determined. Within each parameter, the percent changes for the various brain regions were similar; averaging the percent change from the separate brain regions (±SE) gave the following values: ADC: 18.1 (±5.4); k_io_V: 16.9 (±4.8); k_io_: −4.2 (±4.2); ρ: −7.6 (±3.7); V: 1.1 (±5.2); vi: −8.4 (±1.6). These data indicate that 3 diffusion directions should be used when possible. However, the small magnitude of the diffusion direction effects and their general independence from brain regions suggests that tumor regions would likely not show greater differences than observed here, and most likely would show differences of smaller magnitude due to the random nature and decreased diffusion anisotropy of tumor cell distribution. Hence, results from the trace‐averaged DWI and the DWI with a single diffusion direction (Y) should be comparable and relative changes between regions, and the conclusions of the study, would not be greatly affected. This is consistent with the fact that MADI employs the diffusion tensor magnitude, not its directionality.

In the current study, we noted that an appreciable percentage of voxels, from 0% to 10% depending on the ROI, can match to MADI parameter values that are outside the physiological range. We employed medians of ROIs, rather than means, to minimize their effect. The reason for this is not fully understood, but it is likely that increasing the number of library match combinations (of V, ρ, k_io_) will reduce this problem.

As mentioned above, the Voronoi cell structure model can accommodate a variety of cell shapes, a significant improvement over spherical cell geometry that is employed with some other DWI‐based approaches. Within this context, it is still a simple model which depends on only three variables (V, ρ, k_io_); such simplicity is required for robust matching to the library combinations. Underlying the MADI approach takes into account that the cytolemmal membrane is the primary diffusion barrier in the tissue, with water being in relatively fast exchange within (but not between) the intracellular and extracellular milieu. This enables MADI to measure water transport out of the cells, despite the complexity of the extracellular matrix and intracellular space. MADI considers water transport in all cells within each voxel, including immune, vascular and other cells. That the current study's vi values agree with the brain literature (~0.85) indicates that MADI is properly estimating the intracellular/extracellular space despite its complexity [60].

The utility of the MADI approach in noninvasively assessing tumor cellular metabolism demonstrated in this study suggests there to be potential for assessing other diseases as well. The MADI parameter measurements (Figure 5, Table 1) are sufficiently consistent and reproducible for the detection of subtle differences in cellular metabolism. The MADI approach circumvents issues that limit MRI contrast‐based methods in the brain, and the k_io_V and k_io_Vρ parameters may be particularly sensitive to metabolism in the brain given the high NKA activity and related energy consumption. Novel noninvasive avenues for assessing Alzheimer's disease, Parkinson's disease, epilepsy, stroke, and other diseases are urgently needed, and further studies are required to test the potential utility of MADI in this regard.

In summary, the current study demonstrates the utility of the MADI approach for noninvasively obtaining key cytometric and metabolic information in rat brain with RG2 glioblastoma tumors. Comparison to histology provided validation for MADI sensitivity to ρ. The k_io_V parameter proved useful for tumor detection, with superior image resolution compared to ^18^FDG‐PET. The k_io_Vρ parameter was consistent with increased cellular energy production in tumor. The combination of MADI MRI with ^18^FDG‐PET provided an approach for evaluating the glycolytic energy contribution in the context of overall cellular energy production. The study supports the concept that MADI methodology has the potential to improve upon the current standard of cancer imaging metrics and warrants continued studies to further characterize its utility.

## Author Contributions

M.M.P., C.S.S., R.F.B., X.L.: conception/design. J.W.S., S.M.H., F.D.K., J.S., W.P., X.L., M.M.P.: data acquisition and analysis. E.M.B.: software development. X.L., R.F.B., C.S.S., M.M.P.: manuscript writing/revision.

## Funding

This work was supported by the National Institutes of Health, UL1TR002369. The content is solely the responsibility of the authors and does not necessarily represent the official views of the NIH.

## Conflicts of Interest

C.S.S., E.M.B., and X.L. are inventors on U.S. Provisional Patent Application (No. U.S. 62/482,520) “Activity MRI.”

## Data Availability

All data are available in the main text.
